# Time since Introduction, Seed Mass, and Genome Size Predict Successful Invaders among the Cultivated Vascular Plants of Hawaii

**DOI:** 10.1371/journal.pone.0017391

**Published:** 2011-03-02

**Authors:** John Paul Schmidt, John M. Drake

**Affiliations:** Odum School of Ecology, University of Georgia, Athens, Georgia, United States of America; Duke University, United States of America

## Abstract

Extensive economic and environmental damage has been caused by invasive exotic plant species in many ecosystems worldwide. Many comparative studies have therefore attempted to predict, from biological traits, which species among the pool of naturalized non-natives become invasive. However, few studies have investigated which species establish and/or become pests from the larger pool of introduced species and controlled for time since introduction. Here we present results from a study aimed at quantifying predicting three classes of invasive species cultivated in Hawaii. Of 7,866 ornamental species cultivated in Hawaii between 1840 and 1999, 420 (5.3%) species naturalized, 141 (1.8%) have been classified as weeds, and 39 (0.5%) were listed by the state of Hawaii as noxious. Of the 815 species introduced >80 years ago, 253 (31%) have naturalized, 90 (11%) are classed as weeds, and 22 (3%) as noxious by the state of Hawaii. Using boosted regression trees we classified each group with nearly 90% accuracy, despite incompleteness of data and the low proportion of naturalized or pest species. Key biological predictors were seed mass and highest chromosome number standardized by genus which, when data on residence time was removed, were able to predict all three groups with 76–82% accuracy. We conclude that, when focused on a single region, screening for potential weeds or noxious plants based on a small set of biological traits can be achieved with sufficient accuracy for policy and management purposes.

## Introduction

Extensive environmental and economic damage has resulted from the introduction of invasive exotic plant species [1], [2]. Understanding the process of invasion by exotic species is critical to predicting and preventing the entry of new invasive plants. For this reason, many comparative studies have attempted to predict which species will become invasive, once introduced into a region, from biological traits postulated to be correlated with invasiveness [3], [4]. However, generalization from comparative studies has been hampered by data limitations and ecological complexities that manifest as nonlinearities or interactions in data [5], [6]. Generally, studies with a regional scope, focusing on predicting classes of pest species from the pool of naturalized alien species, have been more successful than studies with universal application [7], [8] (but see [9]). Despite the importance of introduction as a rate-limiting step in the invasion process [10], [11], relatively few studies [12], [13], [14] have included data on the less well-documented, though larger, pool of species which have been introduced and cultivated, but have not naturalized. Even fewer studies [12], [15] have controlled for time between the introduction of a species and naturalization and/or multiplication to pest densities – leaving untested the possibility that many species which are not currently established or invasive may actually naturalize or become pests given sufficient time.

In this study, we investigate the importance of time since introduction and putative biological traits that correlate with a propensity toward naturalization and invasiveness in 7,866 species cultivated on the islands of Hawaii since 1840. Because several studies had indicated a relationship between invasiveness in plants and seed mass [16], [17], [18] and chromosome number or ploidy [19], [20], [21], we compiled, from large online databases, values for both traits as predictor data. To test whether values for these traits relative to closely related species might also be predictive, we calculated genus-standardized values for them where possible. For each class (naturalized species  = 420, weeds  = 141, noxious species  = 39), we then used boosted regression trees [22], [23], a machine learning approach, to 1) quantify the relationship between naturalized, weed or noxious status and minimum years since introduction, and 2) test the prediction that seed mass and genome size alone may be sufficient to classify naturalized and pest species from among introductions to Hawaii with and without controlling for minimum years since introduction.

## Materials and Methods

### Data

The *Annotated Checklist of Cultivated Plants of Hawai'i* (http://www2.bishopmuseum.org/HBS/botany/cultivatedplants/) lists 7,866 cultivated species grown for ornamental and landscape purposes by home gardeners and labels 420 species as naturalized. We combined these data with information on pest species status for Hawaii from the Plants National Database (http://plants.usda.gov/, maintained by the USDA Natural Resources Conservation Service). Noxious species are listed as such by the state of Hawaii, and weeds are designated by the Hawaiian Ecosystems at Risk Project (http://www.hear.org/). Naturalized, weed, and noxious species form nested subsets. While we recognize that these categories combine species which are ecologically disparate, and, therefore, may not closely correspond to the biological groupings recommended by some authorities [24], they are classes which capture degree of current or emerging pest status from the viewpoint of natural resource managers, independent of judgments by us.

Within the Annotated Checklist, the year first collected in Hawaii is recorded for 3,437 (44%) species. Twenty-two species listed as Polynesian introductions were assigned a date coincident with the oldest records (1840). We assume that date of first collection approximates date of introduction rather than date of initial spread. Thus, we subtracted year of first collection from 1999, the latest date in the checklist, to derive a value for minimum years since introduction (∼10–∼150 years) to Hawaii. We compiled data on seed/spore mass and chromosome number both of which are available from large on-line databases. Values for average seed/spore mass were obtained from the *Kew Gardens Seed Information Database* (http://www.kew.org/data/sid) for 1,888 species. Values for highest reported chromosome number for each species were obtained from the *Missouri Botanical Gardens Index to Plant Chromosome Numbers* (http://mobot.mobot.org/W3T/Search/ipcn.html) for 2,221 species. As a means of quantifying whether species were typical or occupiedq extremes within a genus, we calculated a standardized value 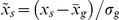 for highest chromosome number and seed mass where 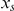 is the measured trait value for species *s*, 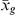 is the mean of all values available from either online database for the genus *s*, and 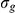 is the genus standard deviation for 1,992 species belonging to a genus where data existed for more than one species. By dividing by the standard deviation, standardization results in a scale-invariant value. We included both raw and standardized values for both seed mass and highest chromosome number in explanatory models. Finally, to control for differences between major groups of vascular plants, we included the categorical variables gymnosperm, fern, and angiosperm as predictors. Data are provided in Table S1.

### Statistical analyses

We used machine learning approaches, specifically boosted regression tree analysis, to develop classification models for each class of invasive plants. Machine learning avoids starting with a data model, instead using an algorithm to learn the relationship between response and predictors [25]. Boosted regression trees which differ from traditional regression methods that produce a single “best” model or tree, relying, instead, on boosting, a technique that combines large numbers of relatively simple models adaptively to optimize prediction [22], [23], [26]. Boosted regression trees have important advantages for improving the analysis of large and complex data sets with many independent variables. Like regularized regression, boosted regression trees provides a robust alternative to traditional approaches such as stepwise variable selection. There is no need for prior data transformation or elimination of outliers. Complex nonlinear relationships can be fit, and interactions between predictors handled automatically. In addition, predictive performance in boosted regression trees is superior to most traditional modeling methods, and despite the complexity of boosted regression trees models, they can be summarized to provide mechanistic insights [22], [23]. All results reported here were obtained using the gbm package in R [27] which has the additional advantages of handling missing data and of allowing weighting of data.

### Model tuning and selection

Because we wished to estimate the expected performance of the model for species that were not used for fitting and to avoid the unrealistically low error rates returned by in-sample comparisons, we randomly divided the data, stratifying by class, into training (75%), and test (25%) sets (22,23). Since the response variable in each model was binary, models were fit using a Bernoulli distribution and logit link. To maximize performance, samples from the positive (minority) class were weighted to compensate for data imbalances, and model parameters were tuned in ten-fold cross-validation such that the minimum number of trees exceeded 1,000 [22], [23]. During fitting, relative importance of predictor variables was calculated as the number of times each variable was selected for splitting, weighted by the squared improvement to the model as a result of each split, averaged over all trees, and rescaled to sum to 100 [28]. Variables with low importance (<2%) were sequentially eliminated if model performance was not reduced. The final model was then used to predict the final 25% holdout test set, providing the performance estimates we report.

Because using the same data for model testing and validation leads to overfitting and deflates the estimated error rate, we used 10-fold cross-validation on a randomly selected 75% training sample for model training. Following model estimation, we used receiver-operator curves (ROC), calculated on the holdout test set, to assess model performance. ROC curves plot the proportion of true positives against the proportion of false positives across the complete range of possible cutoffs. We compared models according to the area under the under the ROC curve (AUC). AUC is a value between 0.5 and 1 which summarizes the probability that a randomly chosen positive case (invasive) has a higher predicted probability than a randomly chosen negative (non-invasive) case. The closer AUC is to 1 the better a model is at discriminating pest from non-pest species.

## Results

The best models predicting naturalization and weed or noxious status relied on three variables: time since introduction, seed mass and highest chromosome number standardized by genus (HCNSG). With these models, we were able to predict the likelihood that a species had naturalized, had been designated a weed, or had been designated noxious with AUC  = 0.88–0.92. Additional predictors (seed mass standardized by genus and highest chromosome number) did not improve model performance. Nor was prediction improved by distinguishing ferns and gymnosperms from angiosperms in the models. The level of prediction achieved using just two biological traits was 0.79 for naturalized, 0.75 for weeds, and 0.82 for noxious.

**Figure 1:**
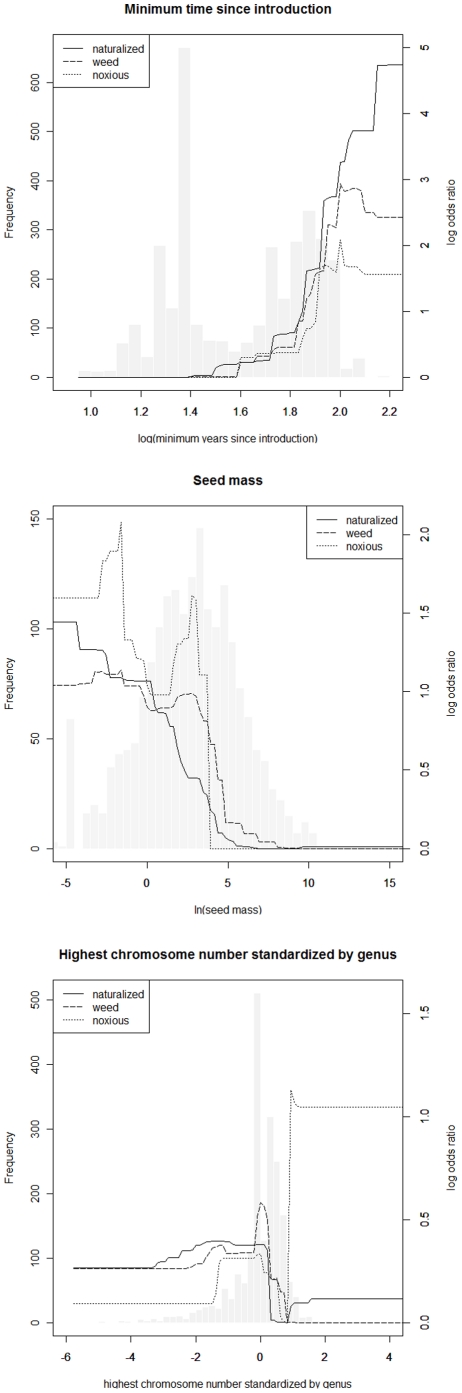
Plots showing the improvement of GBM models as a function of a single predictor [**29**]. The effect of minimum years since introduction, ln(seed mass), and highest chromosome number standardized by genus (HCNSG) on the likelihood of naturalization, weed status, and noxious status, are overlaid on a frequency histogram (left y-axis) of each predictor in the complete data set. Functional values (log odds ratio of naturalized, weed, noxious probability, right y-axis) were standardized by shifting the lowest value to 0.

The relationship between years-since-introduction and naturalized, weed, or noxious status is described by a quasi-logistic curve with a midpoint around 65 years (Fig. 1). Of 815 species introduced >80 years ago (the earliest period for which we have a sizeable sample), 253 (31%) have naturalized, 90 (11%) are weeds, and 22 (3%) are classified as noxious versus 122 (4.4%), 42 (1.5%), and 13 (0.5%) for those introduced <80 years ago. The proportion of species introduced >80 years ago which have naturalized or become pests may represent the leveling off point in the invasion process for Hawaii (assuming there is no relationship between date of introduction and the invisibility of the species introduced), and thus offer the best estimate of base rates of invasion for the system. The probability that a species naturalizes or becomes a pest declines with seed mass (Fig. 1). The smallest seeds or spores (0.001 mg) show the highest probabilities of naturalizing or becoming weeds. Very few naturalized, weed, or noxious species exceed 2.5 g, and few noxious species exceed 2 g although noxious species show a peak just below this threshold. The probability of naturalization is highest for species with average or below average chromosome numbers (relative to congeners; Fig. 1). Weeds show a similar, though bimodal, relationship. However, noxious species exhibit a strikingly divergent pattern: species average or well above average in chromosome number are much more likely to become noxious than species below average.

When models were restricted to a single variable, prediction ranged from 68–82%. Prediction as a function of years-since-introduction was highest for naturalized species (AUC  = 0.82), lower for weeds (AUC  = 0.80), and lowest for noxious species (AUC  = 0.76). HCNSG was the single best predictor of noxious status (AUC  = 0.80), but was the worst predictor of naturalized and weed species (AUC  = 0.69, AUC  = 0.68). Seed mass was similar in predictive performance (AUC  = 0.71–0.74) for all three classes. Prediction as a function of biological variables (seed mass and HCNSG) was greater than prediction as a function of years since introduction for noxious classes (AUC  = 0.82 vs. AUC  = 0.76), but not for naturalization or weeds (AUC  = 0.76 vs. AUC  = 0.82, AUC  = 0.75 vs. AUC  = 0.80) (Table 1, Fig. 1).

**Table 1:** Model performance measured by area under the ROC curve (AUC) values for models of the invader classes as a function of key predictors.

model	model performance (AUC)			num. species
	naturalized	weed	noxious	
full model	0.92	0.91	0.88	4861
years since introduction	0.82	0.80	0.76	3460
seed mass + HCNSG	0.76	0.75	0.82	3180*(864)
HCNSG	0.69	0.68	0.80	2009
**seed mass**	**0.71**	**0.71**	**0.74**	**2077**

*species for which data contains values for either term, number of species with values for both in parentheses.

Scatterplots of the relationship between the three covariates, log(years) since introduction, ln(seed mass), and HCNSG reveal little correlation between them (Figure S1). Given a lack of covariance between explanatory variables, two-way (HCNSG x ln(seed mass), etc.) marginal plots (improvements of the GBM model as a function of a single predictor [29]) of the covariates from the full models suggest a set of interactions between HCSNSG and ln(seed mass) and between log(years) and both HCNSG and ln(seed mass) in the weed and noxious models (Figure S2). Interestingly, whereas seed mass and relative chromosome number appear to interact such that small-seeded species (<1.5 g) with low relative chromosome numbers (value<0) are much more likely to be naturalized or weeds than species with large seeds (>1.5 g) and large relative chromosome numbers (value >0), the interaction is altered for the noxious model such that small-seeded species with large relative chromosome numbers are most likely to be noxious. This pattern is similar in the relationship between time since introduction and HCNSG. Species with high relative chromosome numbers and residence times under 50–80 years are least likely to be weeds, whereas species with similar residence times and low relative chromosome numbers are least likely to be noxious. Finally, small-seeded species (<0.5 g) introduced > 50–80 years prior are much more likely to be either weeds or noxious than large-seeded species introduced more recently.

## Discussion

Despite the socially determined nature of weed and noxious categories, a very low proportion of naturalized, weed or noxious species in the data set, and a large proportion of missing data we were able to predict whether species introduced to Hawaii did or did not become naturalized, weedy or noxious with close to 90% predictive performance. Although clearly facilitated by focusing on ornamental plants within a single geographic region, a striking result was the level of prediction (naturalized  = 0.79, weeds  = 0.75, and noxious  = 0.82) achieved using just two biological traits. Other screening tools – such as those that have been developed for Australia [30], modified for Hawaii [31], and tested successfully in a variety of regions [32] – have been shown to have even higher accuracies. For example, predictive performance (AUC) of >0.90 was achieved for Hawaii and Pacific Islands. Yet, such high levels of prediction required the inclusion of native range size and/or a binary weediness (weedy elsewhere) score [31], in addition to other traits and factors.

Seed mass and HCNSG appear to be highly informative characteristics. Naturalization in plants has been positively related to wind dispersal and negatively related to seed mass [3], [16], [17]. Seed mass may serve as a surrogate for a number of traits important to the population biology of species, such as dispersal ability [33], persistence in the soil [34], time to first reproduction, plant life span and reproductive lifespan [35], seedling survival [36], and number of seeds produced annually per plant [16], [35]. Multiple ploidy levels per species [37], hybridization and polyploidy [17], [38], [39], [40], [41], and DNA content [20], [42] have all been linked to invasiveness in previous studies. However, the relationship appears to be complicated in that weeds have been reported to have smaller DNA C-values than other species yet also more likely to be polyploid [42]. HCNSG, by quantifying relative genome size, may be particularly useful for distinguishing weeds (often associated with smaller genome sizes, small seeds, and rapid development times [42]) from more noxious species, frequently polyploid hybrids with large genomes relative to congeners [19], [21].

Species with seeds or spores below a threshold and with either large or small genomes (relative to congeners) are much more likely to establish and invade once introduced. While seed mass and highest chromosome number (rather than average or typical value for a species) do not appear to be correlated in our data, evidence exists [43] for a complex relationship between genome size and seed mass. In angiosperms, genome size may set a minimum seed mass which increases with increasing genome size, but the maximum seed mass for any given genome size may be determined by other factors [43]. Our results suggest that high chromosome number relative to congeners presumably via recent polyploid events and seeds small enough to facilitate dispersal by wind and vertebrates (<∼1.5 g) increases the likelihood that an introduced species becomes a more serious, or noxious, invader. As an example, *Solidago gigantea* occurs as a diploid, tetraploid, and hexaploid in its native North American range, but is known exclusively as a vigorously rhizomatous tetraploid in its introduced, European, range - supporting the notion that formation of polyploid hybrids may be a key factor promoting colonization and spread of plant introductions [41]. However, in apparent contradiction to the foregoing example is the pattern presented by *Solidago canadensis* which also occurs as a diploid, tetraploid, and hexaploid in its native North American range, but is known as a diploid in its invasive range [44]. Nonetheless, the predictor HCNSG would work equally well in either case given that both species occur as hexaploids, and the ability to form polyploids may represent a kind of phenotypic plasticity or genetic archictecture promoting invasion success within species or genera.

**Figure 2:**
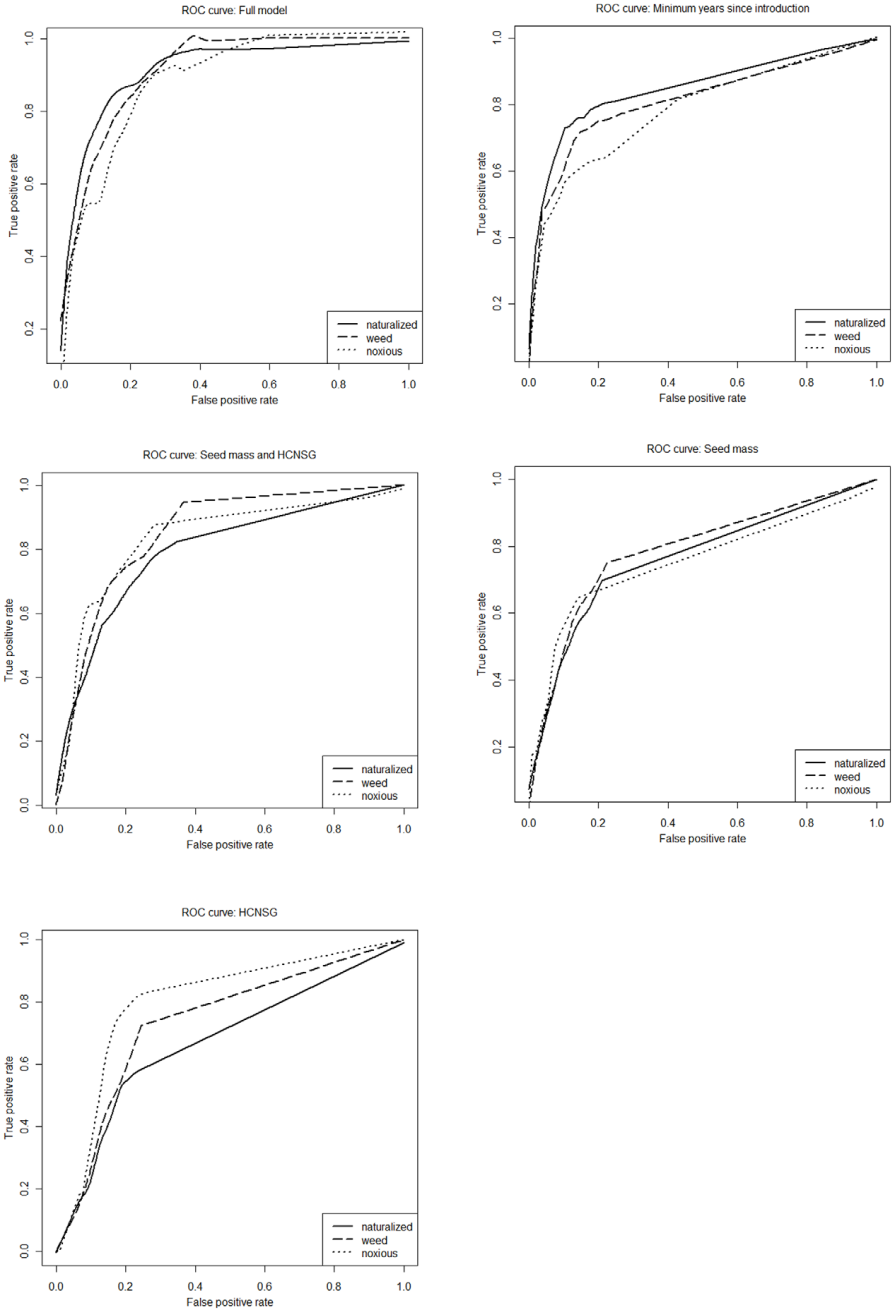
ROC curves showing performance of classifiers for each model. Performance is shown for naturalized, weed, and noxious species from all introduced species as a function of 1) minimum years since introduction, seed mass, and HCNSG, 2) minimum years since introduction, 3) seed mass and HCNSG, 4) seed mass, and 5) HCNSG in discriminating. Increased area between the ROC curve and the diagonal indicates improved classifier performance. Curves were smoothed using the lowess function in the stats package of R.

In addition, we found that prediction as a function of seed mass and HCNSG was greater than prediction as a function of years since introduction for noxious classes, but not for naturalization or weeds. We infer from these results that, while colonization and invasion success is a function of both time and traits conferring the ability to disperse and establish, often in disturbed environments, many species will naturalize or become weeds given enough time, the few species classed as noxious are best predicted by key biological traits (Fig. 2) rather than time since introduction. Moreover, minimum years since introduction was most important in predicting naturalization, but decreased in importance as a predictor of weeds, and was least important as a predictor of noxious species. Together these findings may serve as indirect evidence that pests or serious pests naturalize more quickly - a hypothesis supported by at least one other study [45] but which we were not able to test directly. Finally, we found 1) that weeds, and to a lesser extent, naturalized species, are likely to exhibit values at or below the mean for highest chromosome number relative to congeners, whereas noxious species are likely to be significantly above the mean; and 2) small-seeded species naturalize, and become recognized as pests at a higher rate than larger-seeded species with a threshold at ∼2.5 g. Thus further suggesting that differences between weeds and more serious pests may be related to genome size and phenomena such as hybridization and polyploidy.

Time lags can be distinguished as either the delay 1) between introduction to an area and first spread, or 2) between initial spread and significantly higher population growth, and may result from 3) both [12]. Our estimate of 80 years for the lag time between first introduction and naturalization - time lag type 1 - is low relative to estimates of well over 100 years for Brandenburg, Germany [12] and South Australia [45], yet similar to an estimate for New Zealand [46] where 65 years was the mean residence time for species which were established and spreading. The frequency histogram (Fig. 2) of minimum time since introduction appears bimodal which may be an artifact arising from variability in collecting intensity or represent distinct peaks in the rate of introduction of new plants - over time. If the former is the case, the quasi-logistic relationship between minimum time since introduction and naturalized, weed, or noxious status we report (Fig. 2) with midpoint (mean) at ∼80 years may underestimate lag times. Contrariwise, minimum time since introduction estimates may be too high for species introduced earliest. For those species, the data is right-censored since we have no indication when they were first classed as naturalized, weeds, or noxious. However, we can establish that plants introduced >80 years ago are roughly seven times as likely to be naturalized or pests, implying that many current introductions may eventually naturalize and become pests if base rates can be assumed to be similar for species introduced in different time periods.

Recognizing that species introduced to Hawaii are clearly not a random sample of the global flora, but rather species easily brought into cultivation and expected by horticulturalists to thrive in Hawaii, we emphasize the high level of performance in predicting naturalized, weed, and noxious species as a function of a small set of biological traits. We conclude that the propensity for plants to achieve pest densities appears strongly related to seed mass and HCNSG such that identification of potential weeds and, especially, noxious plants based on these and additional biological traits is possible. Interestingly, in our data set weeds generally have lower chromosome numbers relative to congeners, while the more noxious subset of weeds have much higher relative chromosome numbers. While predictive models are greatly improved by the inclusion of data on time since introduction [15], our results demonstrate that models based on traits alone can perform well providing a crucial tool for identifying likely pests a priori. Risk screening at the level of predictive performance we have demonstrated (naturalized, AUC = 0.75, weeds, AUC = 0.76, noxious, AUC = 0.82) falls within the range (69–79%) likely to be sufficient for cost-effective screening [47]. Furthermore, effective screening of plant introductions is greatly facilitated by the availability of large on-line databases of key plant traits, and, by machine learning algorithms such as GBM which permit analysis of highly incomplete data sets while readily incorporating interactions and complex non-linear relationships.

## Supporting Information

Figure S1 **Scatterplots depicting the relationship between covariates in the complete data set.** Plots are of 1) ln(seed mass) x HCNSG, 2) ln(seed mass) x log(years since introduction), and 3) HCNSG x log(years since introduction).
(TIFF)

Figure S2 **Bivariate plots from full models showing improvements of the GBM model as a function of a single predictor** [**29**] **.** Plots depict the probability that an introduced species is classified as naturalized, weedy, or noxious.
(TIFF)

Table S1 **Hawaii Vascular Plant Introduction Data.**
(XLSX)
