# Supplementary figures and images for: Time since Introduction, Seed Mass, and Genome Size Predict Successful Invaders among the Cultivated Vascular Plants of Hawaii
Source: PLoS One. 2011 Mar 2;6(3):e17391. doi: 10.1371/journal.pone.0017391 (PMC3047568; doi:10.1371/journal.pone.0017391)

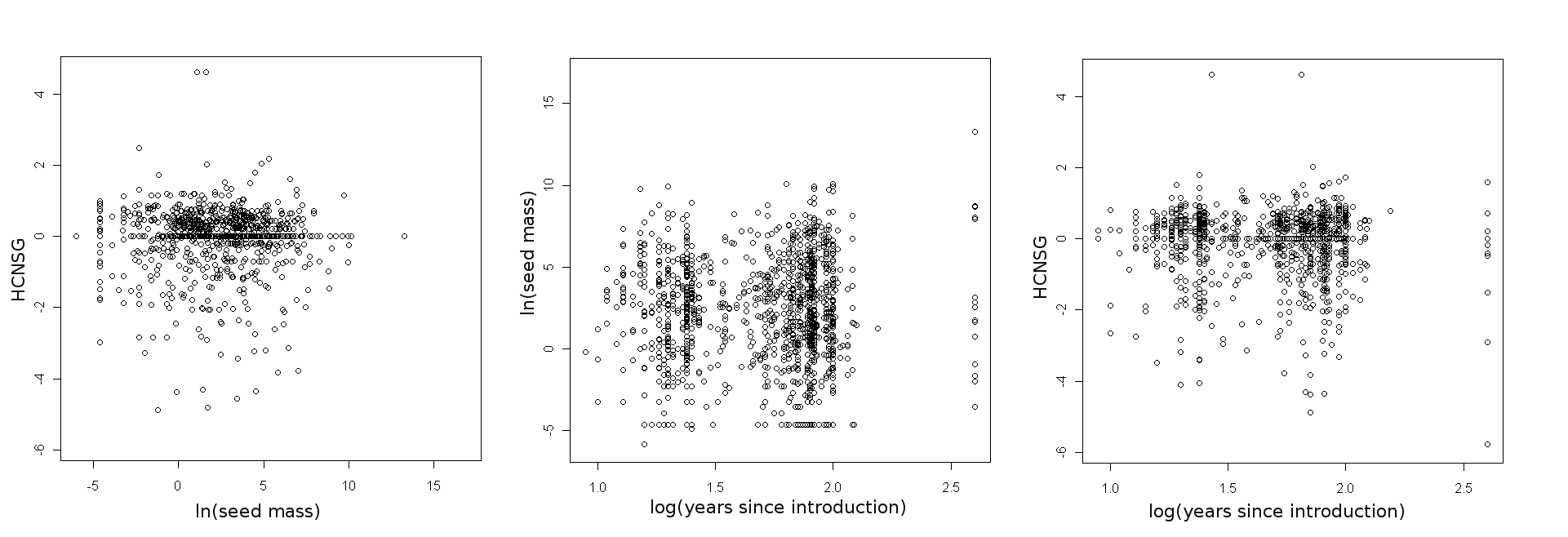

Supplement: Figure S1 — Scatterplots depicting the relationship between covariates in the complete data set. Plots are of 1) ln(seed mass) x HCNSG, 2) ln(seed mass) x log(years since introduction), and 3) HCNSG x log(years since introduction). (TIFF) [file pone.0017391.s001.tiff]

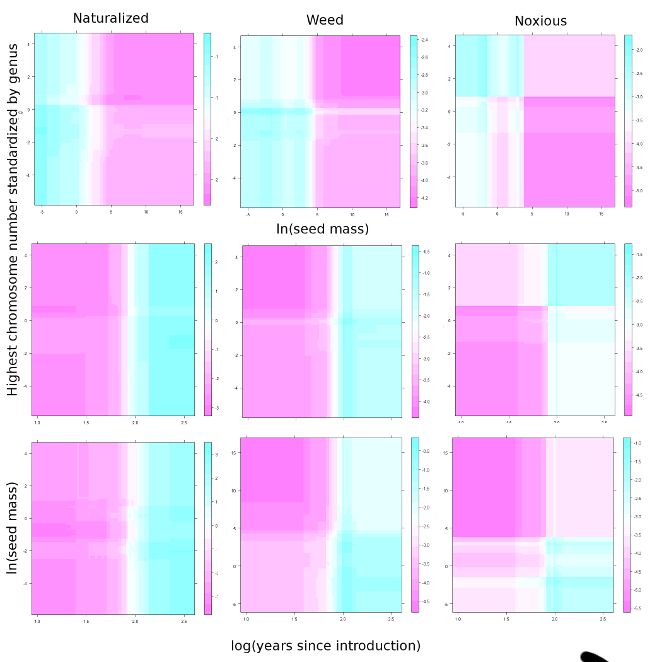

Supplement: Figure S2 — Bivariate plots from full models showing improvements of the GBM model as a function of a single predictor [29] . Plots depict the probability that an introduced species is classified as naturalized, weedy, or noxious. (TIFF) [file pone.0017391.s002.tiff]
